# Updates in the Language of *Histoplasma* Biodiversity

**DOI:** 10.1128/mBio.00181-18

**Published:** 2018-05-08

**Authors:** Pierre Gladieux

**Affiliations:** a BGPI, Université de Montpellier, INRA, CIRAD, Montpellier SupAgro, Montpellier, France

**Keywords:** fungi, gene flow, genomics, pathogens, speciation, systematics, taxonomy

## Abstract

In a recent article, Sepúlveda et al. (mBio 8:e01339-17, 2017, https://doi.org/10.1128/mBio.01339-17) investigated the genetic structure and evolutionary history of the human pathogen *Histoplasma*. Using whole-genome resequencing data, Sepúlveda et al. found that the *Histoplasma* genus is composed of at least four strongly differentiated lineages. Their tour de force is to use a smart combination of population genomic approaches to show that the advanced stage of intraspecific divergence observed within *Histoplasma* does not simply reflect population structure, but instead results from previously unidentified speciation events. The four independently evolving *Histoplasma* lineages are elevated to the species status and assigned names. The newly described species exhibit medically important differences in phenotype, and these findings, therefore, have important epidemiological implications. This work provides a blueprint for phylogenomic species recognition in fungi, opening the way for a new age of enlightenment in which fungal species are diagnosed using highly discriminatory tools within a hypothesis-testing framework.

## FROM PHYLOGENETIC TO PHYLOGENOMIC FUNGAL SPECIES RECOGNITION

The last three decades have witnessed a sustained and concerted attempt to modernize fungal taxonomy, the Latin gibberish used as the language of fungal biodiversity. This modernization effort culminated in the abolishment of the dual nomenclature that permitted the use of different names for the asexual and sexual forms of the same species (1) and translates into a massive weeding out of name redundancy. Besides the dilemma of choosing meaningful and acceptable names, mycologists are also striving to clarify the language of fungal biodiversity by probing into the two main sources of taxonomic ambiguity, uncertainty, and error: the tremendous diversity of fungi and the difficulty of identifying discontinuities in the distribution of genotypes and phenotypes that reflect the existence of independently evolving lineages called species. A great effort is being made to fill in gaps in the fungal tree of life, via large-scale studies of the functional and genetic diversity of fungi in the environment and the sustained production of well-characterized, foundational reference data of fungal genomes (2). Attempts to characterize more finely species boundaries and the structure of fungal biodiversity have also accumulated since the beginning of the millennium. Mycologists tend to abandon methods of species delineation based on *in vitro* measurements of (mostly prezygotic) interfertility and reproductive morphology, given the notorious uncooperativeness of fungi for such experiments (3). Because large-scale crossing studies are formidably challenging in fungi, and because fungal morphology is essentially cryptic, phylogenetic species recognition based on genealogical concordance has emerged as the new “gold standard” for fungal species delineation.

The principle of phylogenetic species recognition schemes is generally to compare nucleotide polymorphisms at typically <10 regions of ca. 500 nucleotides and to identify highly supported monophyletic groups that are concordant across genealogies inferred from different regions and that reflect the existence of reproductively isolated groups (4). The immense advantage of species recognition by genealogical concordance is reproducibility and archivability; the main drawbacks are that reproducibility requires the use of the same set of sequenced loci across studies and that the resolving power of sequenced loci is generally unknown *a priori*. The sequence of diagnostic markers can be readily retrieved from whole-genome sequences, but using genomic data to characterize a few loci is clearly overkill and ignores the mass of information available in genomic sequences. Hence, the challenge is not so much to use genomic data to feed classical approaches to species recognition but to develop methods that make full use of the wealth of information encoded in whole-genome resequencing data. A great benefit of using a genomic approach to identify fungal species is that fungi have relatively small genomes and haploid genetics and as such are highly amenable to genome resequencing. The current flood of fungal genomic data mechanically raises the demand for methods of species identification that can handle such data, but fungal eco-evolutionary genomics lacks a unified and relatively simple framework to elucidate taxonomic subdivisions using population genomics. Working with the human fungal pathogen *Histoplasma*, Sepúlveda et al. (5) deploy the full arsenal of population methods to indirectly measure the presence of gene flow based on resequencing data, and they demonstrate the presence of independently evolving *Histoplasma* lineages that seldom exchange genes, even while coexisting in sympatry.

## SPECIATION AND SEMIPERMEABLE BARRIERS TO GENE FLOW IN *HISTOPLASMA*

*Histoplasma* causes a respiratory infection in humans called histoplasmosis, the most common endemic mycosis in the Americas. Histoplasmosis is also a significant health issue globally, and *Histoplasma* became a genetic model system. The HIV pandemic and increasing use of immunosuppressive medications may have contributed to spread histoplasmosis globally (6), but many aspects regarding the natural and colonization history, environmental reservoirs, and genetic structure of *Histoplasma* remain poorly understood.

In the recent literature, *Histoplasma*—historically the anamorphic classification of the fungus—appears to be preferred over the teleomorphic names *Emmonsiella* and *Ajellomyces*, and there seems to be no debate on this matter. *Histoplasma* was determined to be closely related to other pathogenic dimorphic fungi such as *Coccidioides*, *Blastomyces*, and *Paracoccidioides*, but notable differences in reproductive biology warrant distinct generic names (7). Questions rather concern the cryptic genetic diversity encompassed by the genus, as previous studies based on multilocus Sanger sequencing revealed strong population structure across continents, raising the possibility of undiscovered events of speciation (8, 9). Sepúlveda et al. (5) use a genomic approach to investigate the presence of cryptic species within *Histoplasma*. Their data analysis workflow starts with an unsupervised, model-free approach to analyzing population subdivision. Having identified at least five discrete geographic clusters, the authors set out to explore if the clusters represented distinct phylogenetic species.

One first possible approach to explore the existence of cryptic species based on genomic data is to identify single-copy orthologs, reconstruct genealogical relationships for all orthologs, and use a computational approach that scales to large sets of loci (e.g., see reference 10) to analyze genealogical concordance. However, this approach assumes that single-copy orthologs were correctly identified, which is not always guaranteed. Moreover, the size of orthologous gene sets is expected to decrease with the inclusion of more phylogenetically distant lineages, which can hinder comparisons across studies (11). An alternative approach, used by Sepúlveda et al. (5), is to take the supercontigs as the individual loci. Depending on the completeness of the assembly, one can chose to include all supercontigs in the analysis, or just the largest ones, so that it remains feasible to analyze genealogical concordance by eye. Using the latter approach, Sepúlveda et al. (5) were able to show that the clusters encompassed by *Histoplasma* are reciprocally monophyletic in supercontig genealogies, with genealogical concordance across genealogies inferred for the 10 largest supercontigs. These findings show that previously unidentified speciation events occurred within *Histoplasma* and that the five genetic groups of *Histoplasma* can be referred to as phylogenetic species.

Species diagnostics using species recognition remains an approximate representation of biological diversity in terms of independently evolving lineages. Methods of species recognition are designed to detect lineages that have a long-term history of independent evolution, but none can formally reject the presence of some postdivergence gene flow. Phylogenetic species recognition is based on the premise that conflict among gene genealogies is due to recombination among conspecifics, and the transition from concordance to conflict determines the limits of species. However, the homogenizing effect of gene flow between divergent lineages is expected to only affect a fraction of the genome, not necessarily enough to break concordance among chromosomal genealogies. Hence, the pattern of genealogical concordance uncovered in *Histoplasma* by Sepúlveda et al. (5) can be seen as a proof of the existence of independently evolved lineages called species—warranting the development of species-specific treatments and diagnostic tools—but the finding of phylogenomic species does not preclude more detailed analyses of interlineage gene flow. The data analysis workflow employed by Sepúlveda et al. (5) to explore the extent of gene exchange across newly discovered *Histoplasma* species involved the following steps: (i) quantify the extent of concordance at the whole-genome level and infer the branching order of phylogenetic species using species tree analyses and total evidence genome genealogy; (ii) measure the relative depth of population subdivision by comparing measurements of nucleotide diversity within and between lineages; (iii) assess the plausibility of incomplete lineage sorting as a confounding factor in the analysis of genetic exchanges across lineages; and (iv) test for postdivergence admixture and gene flow using model-based clustering algorithms, phylogenetic tests of gene flow based on numbers of shared derived alleles, and graph-based analyses of population mixtures. Hence, the authors do not merely diagnose species, but they also examine the divergence history of species, quantify the relative depth of divergence between them, and assess hypotheses of interspecific gene flow, and by doing so, they not only corroborate their species delineation work, but also contribute new knowledge about *Histoplasma*’s natural history.

## A BLUEPRINT FOR ASSESSING EVOLUTIONARY INDEPENDENCE IN MICROBIAL EUKARYOTES

The study by Sepúlveda et al. (5) provides a blueprint for the diagnosis of fungal species using whole-genome resequencing data. Their framework both builds on classical methods of species recognition by genealogical concordance and takes full advantage of the inferential power of speciation genomics (12). By incorporating state-of-the-art methods to analyze divergence and gene flow, their approach is more precise, and also more informative, than classical phylogenetic species recognition based on multilocus sequence typing. For instance, speciation genomic analyses can lead to biogeographical hypotheses that would be hardly accessible to nongenomic species recognition by genealogical concordance: species trees and whole-genome analyses of genealogical branching patterns can provide information about the role of vicariance in speciation, and admixture analyses can identify hybrids and show that the species have come into contact in the past. In practical terms, the analytic framework proposed by Sepúlveda et al. (5) has the advantage of being flexible, with the possibility of replacing tools as new approaches become available. Another important practical aspect is that diagnostic assays for epidemiology research and clinical microbial identification can be designed by choosing, in Sepúlveda et al.’s data, loci that recapitulate the supercontig genealogies. One possible limitation though is that taxonomic updates will have to be based on genomic data, as the identification of species is based on the concordance among supercontig genealogies. With species identification implemented in a hypothesis-testing framework, it is still possible that the new species names propelled into the taxonomic space will collide with the conservative inclinations (which generally forms objects with large inertial mass) of the scientists concerned, but the reality of the diagnosed species can hardly be refuted without the inclusion of more data and analysis. The study by Sepúlveda et al. (5) bears the promise that “a new age of enlightenment is at hand in fungal systematics” (13), with mycologists emphasizing hypothesis testing within a speciation genomics framework rather than traditional taxonomic rules and approaches as they endeavor to create a language of fungal biodiversity that captures more accurately the underlying reality of biological discontinuity.
